# Asymmetric Friedel-Crafts Alkylation of Indole with Chalcones Catalyzed by Chiral Phosphoric Acids

**DOI:** 10.3390/molecules14083030

**Published:** 2009-08-13

**Authors:** Arrigo Scettri, Rosaria Villano, Maria Rosaria Acocella

**Affiliations:** 1 Dipartimento di Chimica, Università di Salerno, Via Ponte Don Melillo 84084, Fisciano, Salerno, Italy; E-mail: scettri@unisa.it (A.S.); 2 Istituto di Chimica Biomolecolare-CNR Trav. La Crucca3, Reg. Baldinca 07040, Li Punti, Sassari, Italy; E-mail: rosaria.villano@gmail.com (R.V.)

**Keywords:** asymmetric Friedel-Crafts, indole, chiral phosphoric acids

## Abstract

The reaction of indole with chalcones, to give Michael-type adducts, was found to occur with good efficiency (up to 98% yield) and moderate enantioselectivity (up to 52% e.e.) in the presence of a chiral BINOL-based phosphoric acid. Furthermore, the alkylation products can be obtained in much higher e.e.s after one only crystallization.

## 1. Introduction

The indole moiety represents the main structural feature of a variety of unnatural and natural bio-active products, such as the indole alkaloids [1,2]. In recent years particular attention has been paid to the enantioselective alkylation of indoles with α,β-unsaturated carbonyl compounds [3,4] since the corresponding Michael–type adducts could be considered valuable key-intermediates for the construction of chiral indole architectures. 

It has to be noted that different approaches have been proposed for the Michael-type Friedel-Crafts (F.C.) alkylation of indoles and they involve chiral metal-complexes catalyzed reactions [5,6,7], enantioselective organocatalytic reactions via iminium ions [8,9] and chiral Bronsted Acids [10,11].

**Scheme 1:**
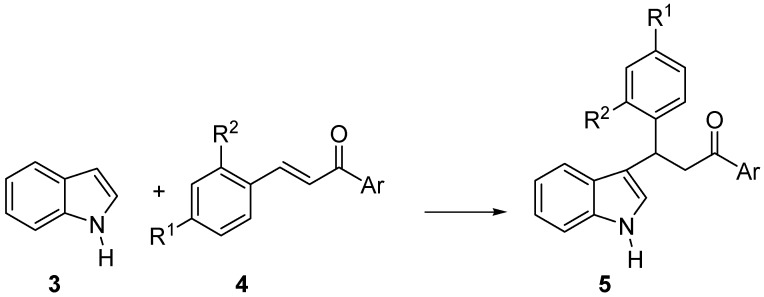
Friedel-Crafts addition of Indole to Chalcones.

**Figure 1:**
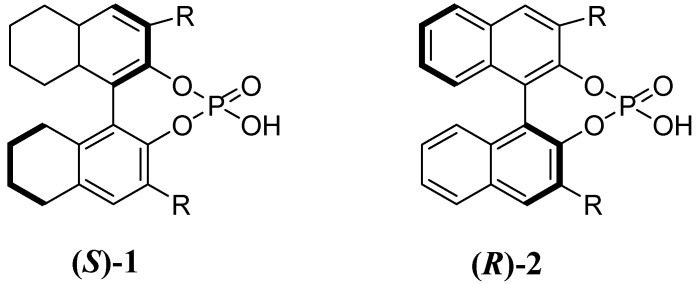
Chiral H_8_-BINOL-based phosphoric acid (***S***)**-1** and BINOL-phosphoric acid (***R***)**-2**.

With regards to chiral Bronsted Acids, good efficiency but rather poor levels of enantioselectivity were observed in the F.C. alkylation of indoles with chalcones when a camphor-based Bronsted acid was used [10], while improved enantiomeric excesses (up to 56% e.e.) were obtained through the use of the H_8_-BINOL-based phosphoric acid of type **1** [11,12] (R= 4-ClC_6_H_4_) (Scheme 1, Figure 1).

Notably, the use of a variety of BINOL-derived Bronsted Acid of type (***R***)**-2** (R= Ph_2_PO, Ph, 4-ClC_6_H_4_, 4-MeC_6_H_4_, 4-PhC_6_H_4_, 2-naphthyl, 3,5-(F_3_C)_2_C_6_H_3,_2,4,6-tBu_3_C_6_H_2_) gave good yields (up to 75%) but rather lower e.e.s (2-35% e.e.).

Taking into account that the different steric and electronic effects of the above cited substituents were found exert a deep influence, both on efficiency and enantioselectivity, we decided to investigate the catalytic properties of the BINOL-derivatives **2a **(R= SiPh_3_), and **2b **(R=4-NO_2_C_6_H_4_), bearing substituents with different electronic and steric properties, in the F.C. alkylation of indole with chalcones.

## 2. Results and Discussion

**Scheme 2:**
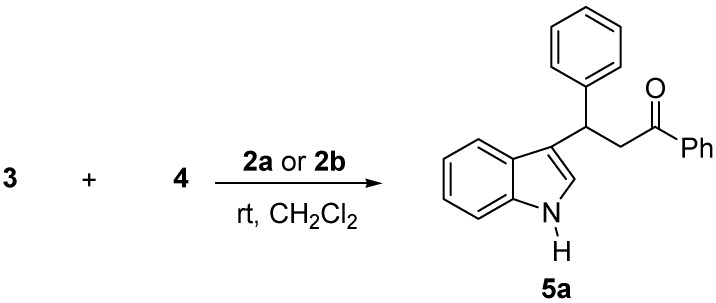
Asymmetric organocatalytic F.C. alkylation of indole **3** with chalcone **4a** catalyzed by **2a** and **2b**.

Initially chalcone **4a **(R^1^=R^2^=H; Ar=Ph) was chosen as a representative substrate and was submitted to reaction with indole **3** under the conditions reported in Table 1 and Scheme 2. Based on the results reported in Table 1, dichloromethane proved a superior solvent with respect to toluene (compare entries 1 and 2), while the organocatalyst **2b** gave better results than **2a**, both in terms of yield and enantioselectivity, provided that more dilute solutions of chalcones **4 **were used (compare entries 3, 4 and 5).

**Table 1:** Asymmetric organocatalytic F.C. alkylation of indole **3** with chalcone **4a**.

Entry	Cat. 2	Reac.Time/h	Yield (%)^a^	e.e.(%)^b^
1^c,d^	**2a **(0.05)	24	25	40
2^e^	**2a **(0.05)	48	25	49
3^d^	**2a **(0.1)	24	35	33
4^d^	**2b **(0.1)	24	60	31
5^e^	**2b **(0.1)	48	82	52
6^e^	**2b **(0.05)	71	40	48
7^e^	**2b **(0.02)	120	25	46

^a^ All the yields refer to isolated chromatographically pure compounds whose structures were confirmed by analytical and spectroscopic data; ^b^ Enantiomeric excess were determined by chiral HPLC; ^c^ In entry 1 toluene was used as solvent; ^d^ 0.5 M solution of chalcones **4** was used; ^e^ 0.15 M solution of chalcones **4** was used.

A lower organocatalyst loading (entries 6 and 7) caused a dramatic drop of the yields and a slight decrease of the e.e.s. It has to be noted that the e.e. of compound **5a**, obtained in entry 5 (52% e.e.) could be enhanced significantly (72% e.e.) by one only crystallization from Et_2_O. The general scope of the procedure was then checked by submitting indole **3 **to treatment with a set of chalcones **4** under the optimized conditions of entry 5, Table 1.

**Scheme 3:**
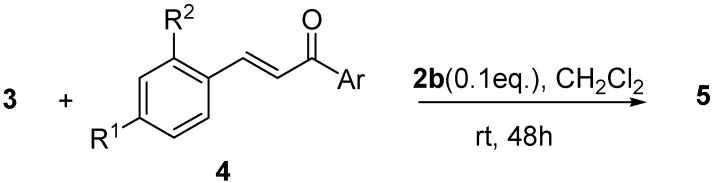
Asymmetric F.C. addition of indole **3** to variously substituted chalcones **4 ** catalyzed by **2b**.

As reported in Table 2, the alkylation of indole was found to take place in moderate to high yields (up to 98%) with variously substituted chalcones while a moderate level of enantioselectivity could be observed for most of the reported starting materials. However, and very interestingly, in several cases the e.e.s of the Michael-type adducts **5** could be again enhanced noticeably (up to 98%) by recrystallization. More simple α,β-unsaturated ketones, such as benzylidene acetone, gave much less satisfactory results since the corresponding alkylation product was isolated in only 15% yield and 30% e.e.

**Table 2:** Asymmetric F.C. addition of indole **3** to variously substituted chalcones **4**.

Entry	Ar	R^1^	R^2^	Product	Yield(%)^a^	Ee(%)^b,c^
1	Ph	H	H	**5a**	82(40)^d^	52(72)
2	Ph	H	Cl	**5b**	98	48
3	Ph	Me	H	**5c**	58(43)^d^	52(70)
4	Ph	OMe	H	**5d**	36	41
5	Ph	NO_2_	H	**5e**	77	46
6	Ph	Cl	H	**5f**	60(30)^d^	52(98)
7	4-ClC_6_H_4_	H	H	**5g**	65(33)^d^	54(97)
8	4-MeC_6_H_4_	H	H	**5h**	44	46
9	4-NO_2_C_6_H_4_	OMe	H	**5i**	73(47)^d^	42(51)

^a^ All the yields refer to isolated chromatographically pure compounds whose structures were confirmed by analytical and spectroscopic data; ^b^ Enantiomeric excess were determined by chiral HPLC; ^c^ Values in parentheses represent the enantiomeric excess observed after crystallization of **5a** and **5c** (from Et_2_O), **5f **and **5g** (from CH_2_Cl_2_/light petroleum ether) and **5**i (from Et_2_O/light petroleum ether); ^d ^Values in parentheses represent the yield after crystallization.

## 3. Experimental

### 3.1. General

All chemicals were purchased from Sigma-Aldrich and used without any further purification. TLC was performed on silica gel 60 F_254_ 0.25 mm on glass plates (Merck) and non-flash chromatography was performed on silica gel (0.063-0.200 mm) (Merck). All ^1^H- and ^13^C-NMR spectra were recorded with a DRX 400 MHz Bruker instrument (400.135 MHz for ^1^H and 100.03 MHz for ^13^C), using CDCl_3_ (δ=7.26 ppm in ^1^H-NMR spectra and δ=77.0 ppm in ^13^C-NMR spectra) as solvent. ^1^H data are reported as follows: chemical shift (δ in ppm), multiplicity (s singlet, d doublet, t triplet, dd doublet of doublets, m multiplet) and coupling costant (*J* in Hz). Optical rotations were measured on a JASCO DIP-1000 polarimeter operating at the sodium D line at room temperature. Concentration is given in g/100 mL. IR spectra were recorded on a Bruker spectrometer. The HPLC analyses were performed with Waters Associates equipment (Waters 2487 Dual λ absorbance Detector) using a CHIRALPAK AD-H column with hexane/isopropyl alcohol mixtures (composition and flow rate as indicated). HPLC methods were calibrated with the corresponding racemic mixtures. Mass spectrometry analysis was carried out using an Waters 4 micro quadrupole electrospray spectrometer. The elemental analyses were calculated with FLASH EA 1112 Thermo equipment. Melting points were determined with an Electrothermal 9100 apparatus. The known compounds have been identified by comparison of spectral data with those reported [8,11].The absolute configureurations of the optically active compounds **5a** was determined on the basis of the measured optical rotation compared with literature values [8,11].

### 3.2. Typical experimental procedure

To a mixture of chalcone (0.125 mmol) and catalyst (0.0125 mmol) 1.2 eq. of indole (0.15 mmol) were added and stirred in dry dichloromethane (0.75 mL) at room temperature. The reaction was monitored by TLC analysis. After 48 hours a saturated aqueous NaHCO_3_ solution (0.75 mL) was added dropwise and the organic layer was extracted in CH_2_Cl_2_, dried over MgSO_4_ and concentrated *in vacuo*. The residue was purified by column chromatography on silica gel in gradient elution with petroleum pther/ethyl acetate to give the pure product.

*3-(1H-Indol-3-yl)-1-phenyl-3-p-tolylpropan-1-one* (**5c**): Yellow solid *m/z* 340 [M+H^+^], 362 [M+Na^+^], 378 [M+K^ +^]; M.p. 91-92 °C; IR (KBr, neat) 3418, 2918, 1687; [α]_D _= (CHCl_3_ c= 0.65, 52% e.e.) = −11.6; HPLC analysis: hexane/*i-*PrOH 98:2, flow rate 0.7 mL/min. t_R_ (major)= 97.3, t_R_ (minor) = 100.3 min.; ^1^H-NMR: *δ* 7.97 (1H, bs), 7.94 (2H, d, *J* = 7.4 Hz), 7.54 (1H, t, *J* = 7.2 Hz), 7.47-7.40 (3H, m), 7.31 (1H, d, *J* = 8.1 Hz), 7.25 (2H, d, *J* = 7.3 Hz), 7.15 (1H, t, *J* = 7.2 Hz), 7.08-6.97 (4H, m), 5.04 (1H, t, *J* = 7.2 Hz), 3.82 (1H, dd, *J* =16.6; 6.8 Hz), 3.71 (1H, d, *J* = 16.6; 7.6 Hz), 2.28 (3H, s); ^13^C-NMR: *δ* 199.2, 141.7, 137.7, 137.2, 136.2, 133.5, 129.6, 129.1, 128.6, 128.2, 127.2, 122.6, 121.9, 120, 111.6, 45.8, 38.4, 21.5; Anal. Calcd for C_24_H_21_NO C, 84.92; H, 6.24; N,4.13; found C, 84.50; H, 6.10; N 4.10.

*3-(1H-Indol-3-yl)-3-(4-methoxyphenyl)-1-phenylpropan-1-one* (**5d**): White solid *m/z* 355 [M^+^], 378 [M+Na^+^], 394 [M+K^ +^]; M.p. 116-118 ° C; IR (KBr, neat) 3424, 2918, 1677, 1180; [α]_D _= (CHCl_3_ c = 0.33, 41% e.e.%) = -12.3; HPLC analysis: hexane/*i-*PrOH 80:20 flow rate, 0.9 mL/min. t_R_ (major) = 24.2, t_R_ (minor)= 26.05 min.; ^1^H-NMR: *δ* 7.98 (1H, bs) 7.93 ( 2H, d, *J* = 7.2 Hz), 7.54 (1H, t, *J* = 7.4 Hz), 7.44-7.41 (2H, m), 7.31 (1H, d, *J* = 8.0 Hz), 7.26 (2H, d, *J* = 8.6 Hz), 7.14 (1H, t, *J* = 7.2 Hz), 7.04-6.98 (2H, m), 6.79 (2H, d, *J* = 8.6 Hz), 5.02 (1 H, t, *J* = 7.2 Hz), 3.79 (1H, dd, *J* = 16.6; 6.6 Hz), 3.75 (3H, s), 3.69 (1H, dd, *J* = 16.6; 7.8 Hz); ^13^C-NMR: *δ* 199.2, 157.9, 137.7, 137.2, 136.8, 133.5, 129.3, 129.1,128.6, 122.7, 121.8, 120.1, 119.9, 114.3, 111.6, 55.7, 45.9, 38.0; Anal. Calcd for C_24_H_21_NO_2_ C, 81.10; H, 5.96; N, 3.94; found C, 81.05; H, 5.89; N, 3.90.

*3-(4-Chlorophenyl)-3-(1H-indol-3-yl)-1-phenylpropan-1-one* (**5f**): White solid *m/z* 359 [M^+^], 382 [M+Na^+^]; M.p. 98-99 °C; IR (KBr, neat) 3445, 2916, 1694, 1219; [α]_D _= (CHCl_3_ c= 0.13, 52%) = -4.6; HPLC analysis: hexane/*i-*PrOH 80:20, flow rate 0.8 mL/min, t_R _(major) = 16.9, t_R_ (minor) = 18.9 min.; ^1^H-NMR: *δ* 8.01 (1H, bs), 7.93 (2H, d, *J* = 7.1 Hz), 7.57-6.98 (11H, m), 5.05 (1H, t, *J* = 7.4 Hz), 3.81 (1H, dd, *J* = 16.8; 6.4 Hz), 3.69 (1H, dd, *J* = 16.8, 8.0 Hz); ^13^C-NMR: *δ* 198.7, 138.3, 136.5, 133.7, 131.5, 129.7, 129.2, 129.0, 128.6, 124.1, 122.9, 122.6, 121.8, 120.1, 119.9, 111.7, 45.5, 38.1; Anal. Calcd for C_23_H_18_ClNO C, 84.89; H, 5.89; N, 4.30; found C, 84.50; H, 5.70; N, 4.25.

*1-(4-Chlorophenyl)-3-(1H-indol-3-yl)-3-phenylpropan-1-one* (**5g**): White solid *m/z* 360 [M+H^+^], 382 [M+Na^+^], 398 [M+K^ +^]; M.p. 101-102 °C; IR (KBr, neat) 3421, 2922, 1685, 1093; [α]_D _= (CHCl_3_ c = 0.33, 54%)= -18; HPLC analysis: hexane/*i-*PrOH 80:20, flow rate 0.8 mL/min. t_R _(major)= 19.9, t_R_ (minor)= 22.3 min; ^1^H-NMR: *δ* 7.99 (1H, bs), 7.85 (2H, d, *J* = 8.4 Hz), 7.45-6.96 (10 H, m), 5.05 (1H, t, *J* = 7.2 Hz), 3.78 (1H, dd, *J* = 16.6; 6.8 Hz), 3.69 (1H, dd, *J* = 16.6; 7.7 Hz); ^13^C-NMR: *δ* 198.0, 146.5, 139.9, 137.1, 135.9, 130.0, 129.4, 129.0, 128.3, 126.9, 122.7, 121.9, 120.0, 119.0, 111.7, 45.6, 38.8. Anal. Calcd. for C_23_H_18_ClNO C, 84.89; H, 5.89; N, 4.30; found C, 84.60; H, 5.75; N, 4.28.

*3-(1H-Indol-3-yl)-3-phenyl-1-p-tolylpropan-1-one* (**5h**): White solid *m/z* 340 [M+H^+^], 362 [M+Na^+^]; M.p. 167-169 °C ; IR (KBr, neat) 3419, 2918, 1704, 1181; [α]_D _= (CHCl_3_ c = 0.33, 46%) = -19; HPLC analysis: hexane/*i-*PrOH 90:10, flow rate 1.0 mL/min. t_R _(major)= 38.1, t_R_ (minor)= 45.4 min; ^1^H-NMR: *δ* 7.97 (1H, bs), 7.84 (2H, d, *J* = 8.0 Hz), 7.44-6.99 (12H, m), 5.07 (1H, t, *J* = 7.1 Hz), 3.79 (1H, dd, *J* = 16.6; 6.9 Hz), 3.69 (1H, dd, *J* = 16.6; 7.6 Hz), 2.39 (3H, s); ^13^C-NMR: *δ* 198.7, 144.3,143.9, 135.2, 133.9, 129.7, 128.9, 128.7, 128.3, 126.7, 122.6, 121.9, 120.1, 119.9, 111.6, 45.6, 38.8, 22.1; Anal. Calcd for C_24_H_21_NO C, 84.92; H, 6.24; N, 4.13; found C, 84.80; H, 6.20; N, 4.10.

## 4. Conclusions

In conclusion, we have developed a Michael-type reaction of indole leading to variously substituted chalcones by using chiral Bronsted Acid **2b** as catalyst. The reaction proceeds with good efficiency and moderate enantioselectivity. The possibility to obtain the alkylation products in much higher e.e.s (up to 98%) after only a single recrystallization provides a practical method to synthesize highly enantiopure 2-indole derivatives. 
